# Linked cycles of oxidative decarboxylation of glyoxylate as protometabolic analogs of the citric acid cycle

**DOI:** 10.1038/s41467-017-02591-0

**Published:** 2018-01-08

**Authors:** Greg Springsteen, Jayasudhan Reddy Yerabolu, Julia Nelson, Chandler Joel Rhea, Ramanarayanan Krishnamurthy

**Affiliations:** 1 Department of Chemistry, Furman University, Greenville, SC 29613 USA; 2 NSF/NASA Center for Chemical Evolution, Atlanta, GA 30332 USA; 3 Department of Chemistry, The Scripps Research Institute, La Jolla, CA 92037 USA

## Abstract

The development of metabolic approaches towards understanding the origins of life, which have focused mainly on the citric acid (TCA) cycle, have languished—primarily due to a lack of experimentally demonstrable and sustainable cycle(s) of reactions. We show here the existence of a protometabolic analog of the TCA involving two linked cycles, which convert glyoxylate into CO_2_ and produce aspartic acid in the presence of ammonia. The reactions proceed from either pyruvate, oxaloacetate or malonate in the presence of glyoxylate as the carbon source and hydrogen peroxide as the oxidant under neutral aqueous conditions and at mild temperatures. The reaction pathway demonstrates turnover under controlled conditions. These results indicate that simpler versions of metabolic cycles could have emerged under potential prebiotic conditions, laying the foundation for the appearance of more sophisticated metabolic pathways once control by (polymeric) catalysts became available.

## Abstract (web summary)

The citric acid cycle (TCA) is a fundamental metabolic pathway to release stored energy in living organisms. Here, the authors report two linked cycles of reactions that each oxidize glyoxylate into CO_2_ and generate intermediates shared with the modern TCA cycle, shedding light into a plausible TCA protometabolism.

## Introduction

**Fig. 1:**
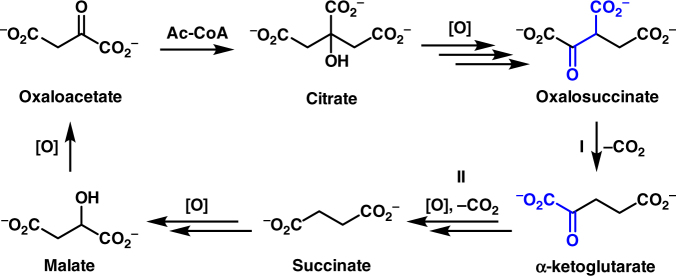
Oxidation and decarboxylation within the modern TCA cycle. A net transformation of Ac-CoA into CO_2_ occurs through a decarboxylation of a β-ketoacid (I) and an oxidative decarboxylation of an α-ketoacid (II)

The establishment of protometabolic cycles has important consequences for the origins of life^1–4^. It has been hypothesized by Leslie Orgel that “If complex cycles analogous to metabolic cycles could have operated on the primitive Earth before the appearance of enzymes or other informational polymers, many of the obstacles to the construction of a plausible scenario for the origin of life would disappear^5^”. The modern metabolic citric acid cycles (TCA and reverse-TCA) are the most investigated in this context^4, 6–10^. The TCA cycle is comprised of a series of enzyme-catalyzed transformations enabling the net oxidation of an acetyl group into two molecules of carbon dioxide^11^. The exergonicity of this conversion is coupled to the production of reduced nicotinamide and flavin coenzymes and nucleotide triphosphates directly, and into aspartate and glutamate synthesis by the reductive amination of TCA cycle intermediates oxaloacetate and α-ketoglutarate, respectively. These amino acids then serve as scaffolds for eight additional biological amino acids, as well as pyrimidine nucleobases^12^. The cycle begins with the aldol addition of acetyl-CoA to oxaloacetate. The product, citrate, is reorganized to enable the decarboxylation of a β-ketoacid (oxalosuccinate, I, Fig. 1), and the oxidative decarboxylation of an α-ketoacid (α-ketoglutarate, II), effectively oxidizing the acetyl-CoA into two equivalents of CO_2_, with the regeneration of oxaloacetate from malate.

However, attempts at using TCA pathways as a template for prebiotic reactions have not yet yielded sustainable non-catalytic cycles^5, 13–19^. Instead of this top-down endeavor to replicate modern biochemistry, we relied on a bottom-up approach by starting with simple abiotic carboxylates including glyoxylate, the smallest α-ketoacid, and malonate, the simplest α-dicarboxylate. In place of modern redox coenzymes, we used hydrogen peroxide (H_2_O_2_), a product of the photochemical oxidation of water^20^. We then focused on reaction types that are fundamental to modern TCA cycle metabolism: aldol addition and oxidative decarboxylation. With this strategy, we discovered two linked cycles of reactions that each oxidize glyoxylate into CO_2_, and generate intermediates that are shared with the modern TCA cycle. Both cycles proceed uncatalyzed at mild temperature and neutral pH, with one cycle demonstrating multiple turnover with sequential feeding of source materials (glyoxylate and H_2_O_2_). In a similar functional role as the TCA cycle, the protometabolic cycle intermediates also serve as a source of amino acids.

## Results

### Reaction cycles

**Fig. 2:**
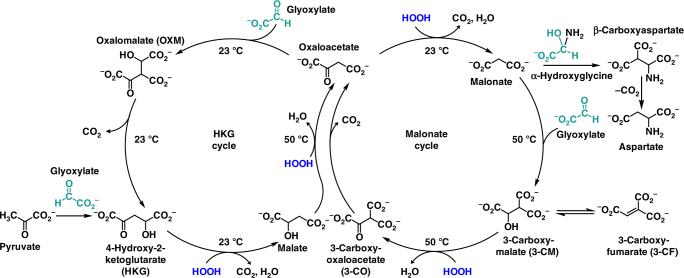
Two abiotic cycles each oxidize glyoxylate into CO_2_ with the regeneration of oxaloacetate. All reactions of both the HKG and Malonate cycles progress significantly in hours at pH values 7–8.5 at the listed temperature of 50 °C or 23 °C

The two abiotic cycles demonstrated in this work (Fig. 2) fundamentally employ the same chemistry, spontaneous decarboxylations of β-ketoacids and oxidative decarboxylations of α-ketoacids, while generating intermediates that are common with the TCA cycle (oxaloacetate and malate, Fig. 2). Both abiotic cycles use glyoxylate as the C2 carbon source rather than acetate (Ac-CoA). However, unlike the TCA cycle, no transformations beyond an aldol addition and an alcohol oxidation are required to enable the decarboxylation chemistry, thus greatly simplifying the overall pathway from nine steps in the TCA cycle to four steps in each abiotic protometabolic cycle. The series of abiotic reactions can be initiated by an aldol addition of glyoxylate with either oxaloacetate, pyruvate or malonate. The only reactants required in either cycle (4-hydroxy-2-ketoglutarate (HKG) or Malonate, Fig. 2) are glyoxylate and H_2_O_2_, and all reactions proceed at mild pH (7.0 to 8.5) and temperature (≤50 °C).

### Malonate cycle chemistry

**Fig. 3:**
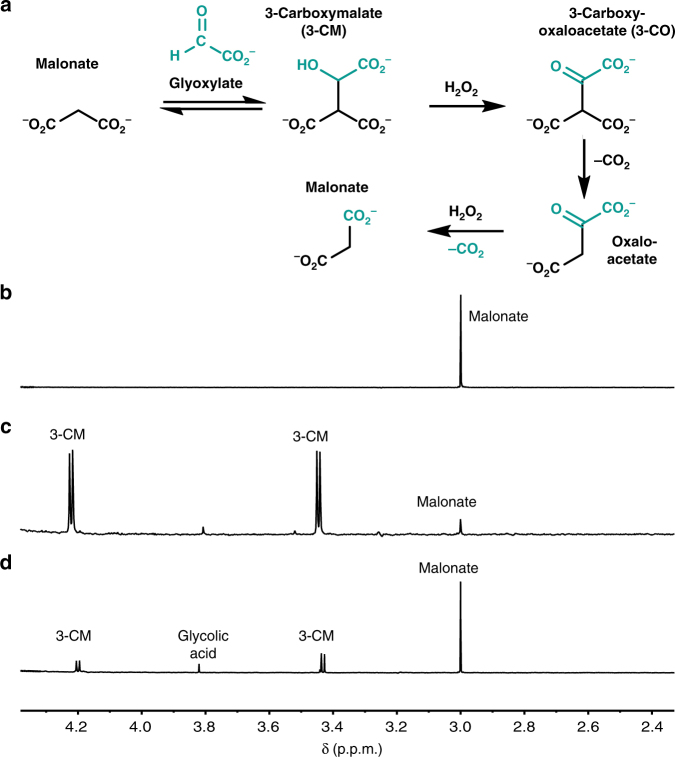
Progression around the Malonate cycle. **a** The reaction pathway of the Malonate cycle, with glyoxylate-sourced atoms in turquoise. **b** ^1^H NMR (in D_2_O) of a reaction aliquot from 90 mM disodium malonate in 1.0 M aq. bicarbonate buffer, pH 8.4. **c** A total of 3 eq. of glyoxylate was added and the reaction was heated for 24 h at 50 °C. **d** A total of 30 eq. of H_2_O_2_ was added and the reaction continued at 50 °C for an additional 48 h. The production of malonate through the oxidative decarboxylation of oxaloacetate, apart from the retro-aldol of 3-CM, was demonstrated by ^13^C labeling, as will be shown below

In a reaction of 90 mM malonate with 3 equivalents (eq.) of glyoxylate at 50 °C, an aldol addition generates 3-carboxymalonate (3-CM; Fig. 2, Malonate cycle, Fig. 3a). At pH 8.4 in bicarbonate buffer, this reaction is nearly quantitative, producing ≥98% 3-CM in 24 h (Fig. 3b, c, Supplementary Table 1). After addition of 30 eq. of H_2_O_2_, and heating at 50 °C for an additional 48 h, regeneration of malonate from the 3-CM intermediate is observed (51% yield, Fig. 3c, d, Supplementary Table 1). This suggested that a series of one-pot oxidation and decarboxylation reactions of 3-CM had taken place, proceeding by oxidation to 3-CO (3-Carboxy-malate), decarboxylation to oxaloacetate, then oxidative decarboxylation of oxaloacetate back to malonate (Fig. 3a, Supplementary Fig. 1). The implication that oxaloacetate, one of the long sought-after molecules in protometabolic (reductive citric acid cycle) scenarios^5, 7, 15^, with little success so far, is formed as a putative intermediate in a plausible prebiotic setting was exciting. Moreover, oxaloacetate has the reaction potential (similar to malonate) to act as a nucleophile and undergo aldol addition with glyoxylate, creating opportunities for diversifying the protometabolic pathways.

### HKG cycle chemistry

**Fig. 4:**
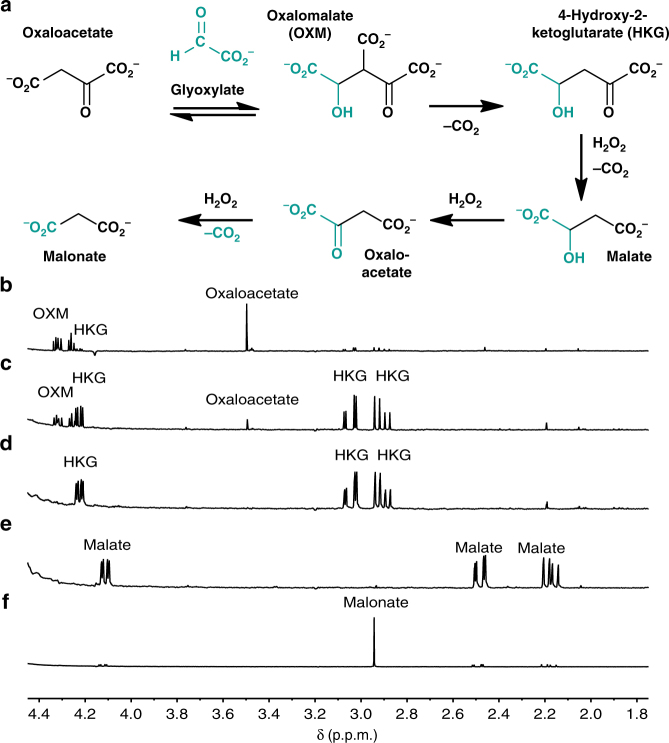
Progression around the HKG cycle. **a** The reaction pathway of the HKG cycle. **b** ^1^H NMR (in D_2_O) of a reaction aliquot from 90 mM oxaloacetate in 1.0 M aq. phosphate buffer, pH 7.0, with 1.2 eq. of glyoxylic acid, stirred at 23 °C for 5 min, **c** for 60 min, and **d** for 180 min. **e** A total of 2 eq. of H_2_O_2_ was added and the reaction was stirred for additional 20 min. **f** An additional 15 eq. of H_2_O_2_ was added and the reaction was heated at 50 °C for 24 h

We thus investigated the aldol addition of oxaloacetate with glyoxylate (Fig. 2, HKG Cycle, Fig. 4a). A reaction containing 90 mM oxaloacetate and 1.2 eq. of glyoxylate at pH 7.0 produces the aldol product oxalomalate (OXM) within minutes at 23 °C (Fig. 4b). A concurrent decarboxylation of OXM to HKG is complete within 3 h, with a two-step yield of ≥98% (Fig. 4b–d). Subsequent addition of 2 eq. of H_2_O_2_ to the thus produced HKG results in a quantitative oxidative decarboxylation to form malate in under 30 min (Fig. 4e, Supplementary Table 1). The oxidation of malate’s secondary hydroxyl to a keto group is rate and yield limiting, as is the oxidation of 3-CM in the Malonate cycle. The addition of 15 more eq. of H_2_O_2_ and heating at 50 °C for 24 h produces 55% malonate (Fig. 4f, Supplementary Table 1). The inclusion of a Lewis acid catalyst (ferrous sulfate, 0.25 eq.) accelerates the reaction and produces a similar result in 3 h rather than 24 h (Supplementary Table 1).

### Cycle tolerance and robustness

**Fig. 5:**
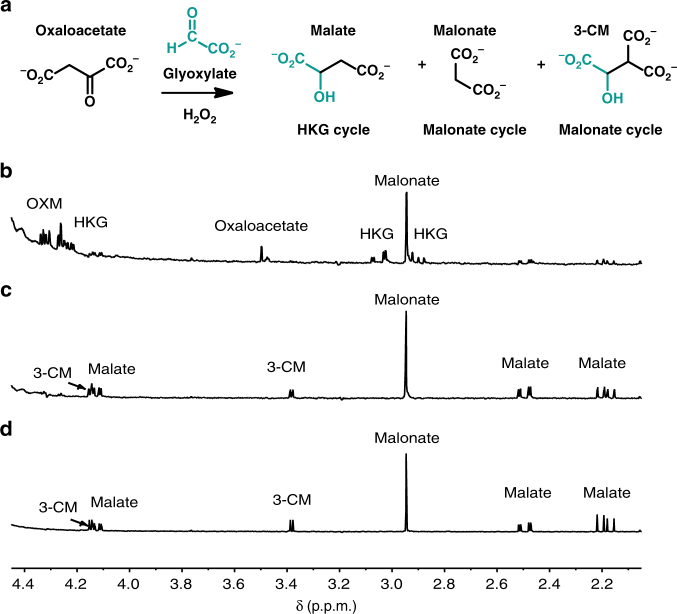
Both HKG and Malonate pathways progress in the presence of glyoxylate and H_2_O_2_. **a** Intermediates of both pathways are detected, OXM, HKG, and malate from the HKG cycle, and malonate and 3-CM from the Malonate cycle. **b** ^1^H NMR (in D_2_O) of a reaction aliquot from 90 mM oxaloacetate, 1.25 eq. glyoxylic acid, with 5 eq. of H_2_O_2_ added over 1 h, in 1.0 M phosphate buffer, pH 7.0, at 23 °C for 10 min, **c** for 30 min, and **d** for 48 h

Both the Malonate and HKG cycles proceed in a single pot with alternating additions of glyoxylic acid, as the carbon source, and H_2_O_2_ as the oxidant. Starting from oxaloacetate, initial addition of glyoxylate induces the HKG cycle, while initial addition of H_2_O_2_ begins the Malonate cycle. The first reaction in either direction of the abiotic cycle starting from oxaloacetate is quantitative at 23 °C (Supplementary Table 1). Co-administration of both glyoxylic acid and hydrogen peroxide (added slowly dropwise) to oxaloacetate induces both cycles concurrently (Fig. 5). When added stoichiometrically all at once, H_2_O_2_ reacts with glyoxylate as quickly as with other cycle α-ketoacids to produce formate (Supplementary Fig. 2).

The robustness of the system (Fig. 2) is demonstrated by the three distinct reactions that oxaloacetate undergoes under these conditions. All three reactions feed into (or back into) the protometabolic cycles, rather than producing a complex array of compounds, as might be expected from an uncatalyzed reaction network. First, the oxidative decarboxylation of oxaloacetate produces malonate, which reacts with glyoxylate to form 3-CM. Second, the aldol addition of oxaloacetate with glyoxylate produces OXM, which decarboxylates to HKG, and third, the non-oxidative decarboxylation of oxaloacetate generates pyruvate. Although, in other contexts, pyruvate might act as a leak and funnel off reactants in an irreversible manner^5^, here pyruvate is recovered by reaction with glyoxylate to regenerate HKG. Pyruvate, obtained from other sources^7, 21^, could also act as an entry point into the protometabolic cycles. After the aldol addition of pyruvate with glyoxylate (Supplementary Fig. 3, Supplementary Table 1), oxidation with H_2_O_2_ produces malate and then oxaloacetate in situ, followed by oxidation to malonate. This in situ generation of oxaloacetate from pyruvate has been a long-sought and elusive goal in prebiotic chemistry^5, 7, 15^. It is, in sum, an α-carboxylation of pyruvate, a critical anabolic pathway in modern biology, which resupplies TCA cycle intermediates (anaplerosis)^22^.

No reagents beyond glyoxylate and hydrogen peroxide are required to progress in either cycle (Fig. 2), and both cycles are tolerant to changes in buffer and pH. Although the aldol addition and decarboxylation of oxaloacetate to produce HKG proceeds optimally at pH 7 (Supplementary Fig. 4, Supplementary Table 1), the reaction progresses cleanly, though more slowly, at lower (5.5) and higher pH (8.0). At a pH of 10.5, HKG is still the dominant product, though other addition and condensation reactions are competitive (Supplementary Fig. 4d). No significant differences in HKG formation were noted when switching between phosphate (pH 2–12) and carbonate (pH 8–10.5) buffers when observed at similar pH values (Supplementary Figs. 4, 5). The oxidation of malate to malonate by H_2_O_2_ is most favorable at pH values from 6 to 8, but is significantly slowed in highly acidic (pH 2) and basic environments (pH 12) (Supplementary Figs. 5, 6). In the Malonate cycle, the aldol addition of glyoxylate to malonate produces 3-CM at pH values from 5.5 to 10.5, with increased reactivity under more acidic conditions (Supplementary Fig. 7). The reaction cycles have been shown to operate at ≤50 °C. While the linked cycle of reactions can all be potentially run at 23 °C, some of the slower steps such as the oxidation of the secondary alcohols (malate and 3-CM) were conducted at 50 °C in order to complete the reactions within a reasonable period of time (24–48 h).

The reaction conditions are quite mild as compared to those required for non-enzyme-catalyzed TCA and reverse-TCA (rTCA) steps that use canonical intermediates. Examples in the literature include three non-contiguous steps in the rTCA: oxaloacetate to malate (75%), fumarate to succinate (95%) and succinate to α-ketoglutaric acid (2.5%), carried out in ultraviolet (UV)-irradiated solution of aqueous ZnS colloid^8, 23, 24^. The oxidative decarboxylation of isocitric acid to α-ketoglutaric acid was observed in the presence of Na^+^-montmorillonite and O_2_^25^. Trace amounts of citric, succinic, aconitic, oxaloacetic and fumaric acids were detected in reactions of pyruvate in basic aqueous cyanide^21^. Multiple oxidation and isomerization reactions were observed to interconvert TCA intermediates in the presence of aqueous peroxydisulfate and iron disulfide^17^. An aldol addition of oxaloacetate to pyruvate^26^, or a second equivalent oxaloacetate^27^, produces citric acid through oxidative decarboxylation in the presence of aqueous peroxide. Recently, a non-enzymatic promotion of multiple reactions of the rTCA cycle in consecutive sequence mediated by Zn^2+^, Cr^3+^ and Fe^0^ has been demonstrated in 1 M aq. HCl and 1 M aq. H_2_SO_4_ at 80–140 °C^19^.

### Cycle turnover

**Fig. 6:**
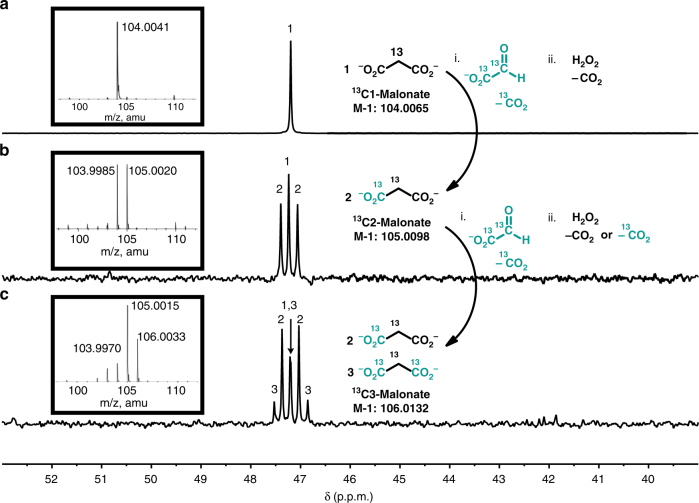
The progression from mono- to di- to tri-^13^C-labeled malonate indicates two full iterations of the Malonate cycle. **a** ^13^C NMR (in D_2_O) of a reaction aliquot from 2.0 ml of 200 mM (2-^13^C1)malonic acid in 1.0 M aqueous NaHCO_3_. **b** A total of 2 eq. of (1,2-^13^C2)glyoxylic acid was added, and the reaction was stirred at 50 °C for 3 h. Then, 30 wt% aqueous H_2_O_2_ was subsequently added to the solution at a rate of 40 eq./h, and the reaction was stirred at 50 °C for 10 h. **c** The reaction volume was reduced under vacuum back to 2.0 ml, and the procedure was repeated starting from the addition of (1,2-^13^C2)glyoxylic acid

The limited number of species (i.e., the sparseness of the network)^28^ and the multiple pathways that lead back to them offer a simpler and alternative approach to a protometabolic network, as opposed to creating a large complex network of reactions and then pruning them in a prebiotic context^15^. The cyclic systems developed here also provide the opportunity to address a key challenge and criticism of previously proposed abiotic reaction cycles^5^, namely the absence of a sustained turnover of reactions. The single-pot regeneration of malonate suggested that a cycle of reactions with turnover could be run starting from malonate with only the sequential addition of glyoxylate followed by oxidant. Turnover of the Malonate cycle was demonstrated using ^13^C-substituted substrates. Singly ^13^C-labeled (2-^13^C1)malonic acid was reacted with doubly ^13^C-labeled (1,2-^13^C2)glyoxylic acid, and oxidized with H_2_O_2_ to complete a full iteration of the cycle, regenerating malonate as a di-^13^C-labeled species (Fig. 6a, b). A second iteration of the cycle with doubly ^13^C-labeled (1,2-^13^C2)glyoxylic acid followed by oxidation produces 50% tri-^13^C-labeled and 50% di-^13^C-labeled malonate (Fig. 6c). The carbon-13 nuclear magnetic resonance (^13^C NMR) spectra of the central carbon of tri-^13^C-labeled malonate show the expected splitting pattern resulting from one and two additional ^13^C labels (Fig. 6b, doublet (2), Fig. 6c, triplet (3)). HRMS high resolution mass spectrometry) data of each stage of the reaction cycle is also consistent with cycle turnover (Fig. 6a–c, insets). A similar demonstration for the HKG cycle is not possible; the oxidation of malate to oxaloacetate proceeds quickly to malonate, thus sending material from the HKG cycle into the Malonate cycle, rather than returning to and progressing through multiple HKG iterations.

### Amino acid synthesis

**Fig. 7:**
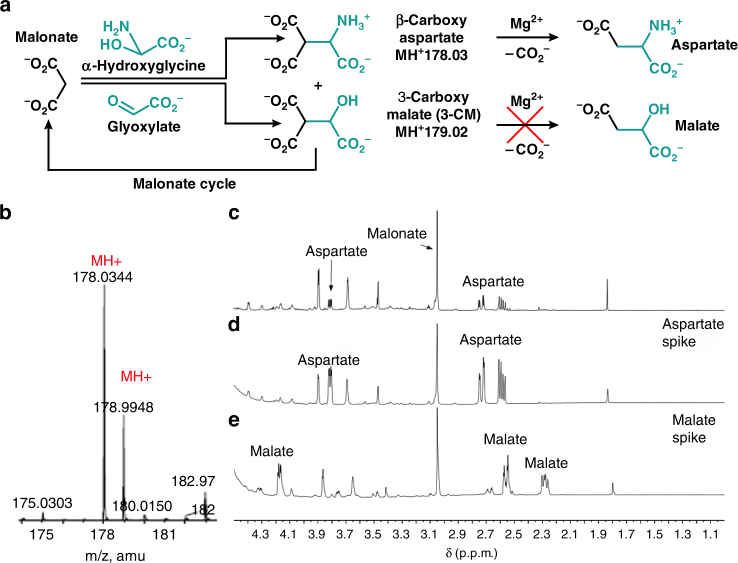
Aspartate synthesis from malonate, glyoxylate, and ammonia. **a** Malonate in the presence of a 1:1 mixture of glyoxlate and α-hydroxyglycine produces 3-CM and β-carboxyaspartate. The β-carboxyaspartate selectively decarboxylates to aspartate. **b** HRMS of a reaction aliquot from 0.5 M malonate and 1 eq. of both α-hydroxyglycine and glyxolate in a pH 6.8 bicarbonate buffer stirred at 23 °C for 5 days. **c** Subsequent addition of 1 eq. of MgCl_2_ and stirring at 50 °C for 5 h produces aspartate, but not malate. **d**, **e** Aspartate and malate spikes, respectively, to **c**

The overlap between constituents of the protometabolic cycles and biologically relevant compounds of modern metabolism provides an opportunity to identify additional significant abiotic pathways. For example, a synthesis of the amino acid aspartate from malonate has been identified here, which proceeds under conditions compatible with cycle chemistry. Malonate reacts with α-hydroxyglycine (generated from NH_3_ and glyoxylate) to produce β-carboxyaspartate, which decarboxylates to aspartate in the presence of Mg^2+^ (Fig. 7, Supplementary Fig. 8, Supplementary Table 1). The production of β-carboxyaspartate proceeds at neutral pH and mild temperature (room temperature) over 5 days. In the presence of 1 eq. of Mg^2+^, a subsequent decarboxylation to aspartate is complete in 5 h at 60 °C in a 50% overall (two-step) yield. The reaction pathway to aspartate requires no additional reagents beyond ammonia (α-hydroxyglycine is produced by the trapping of ammonia with glyoxylate)^29^, and magnesium. A reaction of malonate with a mixture of glyoxylate and α-hydroxyglycine (1:1) results in a mixture of 3-CM and aspartate (Fig. 7), suggesting that the presence of NH_3_ is not detrimental to the overall reaction cycle and instead augments the protometabolic reaction pathways by constructive participation to produce aspartic acid when ammonia is available. The synthesis of aspartate provides a potential function for the protometabolic cycles in generating the building blocks of proteinaceous catalysts, which could in turn influence some of the pathways in the protometabolic cycle.

Links to other chemical systems may also be possible with increasing chemical complexity, as aspartate is a precursor in pyrimidine biosynthesis and in a potential abiotic pyrimidine synthesis^30^. We note with caution, however, that similarities between the protometabolic cycles and the evolved biochemical pathways do not imply that there must be a historical link between them. It has been pointed out that chemical equivalences “do not necessarily indicate an evolutionary continuity between prebiotic chemistry and biochemical pathways^31^”.

### Cycle intermediates and feedstocks

The simple source molecules comprising the protometabolic cycles, including glyoxylate, malonate, pyruvate and H_2_O_2_, raises the question of their availability, and compatibility, in the context of prebiotic chemistry. Glyoxylate^32, 33^ and pyruvate^7, 9, 21, 34^ are small α-ketoacids, whose abiotic production has been noted through the oxidation of hydroxyacids, the oxidative deamination of amino acids, the oxidation of acetic acid in aqueous aerosols, the hydrothermal carbonylation of iron sulfide minerals and delivery by meteorites. H_2_O_2_ is a product of the photochemical oxidation of water catalyzed by pyrite^20^; it has been detected in multiple extraterrestrial environments^35, 36^, and mechanisms for its production and concentration on the early earth have been proposed^37^. As shown herein, malonate can be produced from glyoxylate, pyruvate and H_2_O_2_; it is also generated by the UV irradiation of aqueous acetic acid^38^, and as an end-product of the H_2_O_2_-dependent oxidation of larger polycarboxylates^39^. Although the cycles demonstrated in this work are partially tolerant to the co-administration of both hydrogen peroxide and glyoxylate, an early earth environment that plausibly produces both glyoxylate and H_2_O_2_ amenable to these protometabolic cycles has not been identified and thus remains open to further exploration.

## Discussion

In summary, two linked reaction sequences starting from simple carboxylates and α-ketoacids have been demonstrated to operate under plausible prebiotic conditions. The resulting protometabolic cycles each mimic a simplified version of the TCA cycle; a C2 organic source (glyoxylate) undergoes an aldol addition, a decarboxylation of a β-ketoacid and an oxidative decarboxylation of an α-ketoacid to generate two equivalents of CO_2_. In addition to reaction types, the abiotic cycles feature the intermediates malate and oxaloacetate, and the feedstock pyruvate, all in common with the modern TCA cycle. The dependence on glyoxylate also recalls the modern Glyoxylate cycle, an anabolic TCA cycle variant, which may suggest an important evolutionary link between glyoxylate and the TCA cycle—a possibility that has been raised and discussed elsewhere^40, 41^. The incorporation of ammonia in the Malonate cycle, as the carbinolamine of glyoxylate, leads to the synthesis of aspartic acid. The reaction chemistry achieves a net carboxylation (pyruvate to oxaloacetate) without requiring CO_2_ /ATP, and a net reductive amination (oxaloacetate to aspartate) without requiring a reductant. Alternative transformations such as these, which functionally substitute for enzyme-dependent modern metabolic pathways, may enhance the plausibility of an early protometabolism, including in environments that are not considered amenable to modern biochemistry. When combined with opportune co-emergence and involvement of polymeric catalysts, the transformation of these pathways into more complex metabolic reactions cycles with increased functionality may be an expected outcome.

## Methods

### HKG cycle

Oxaloacetate to HKG: A total volume of 119 mg of oxaloacetic acid (0.90 mmol, 90 mM) was dissolved in 9.9 ml of a 1.0 M aq. pH 7 sodium phosphate buffer in a 20 ml vial with a small stirbar. Then, 119 µl (160 mg) of 50 wt% glyoxylic acid in H_2_O (1.2 eq.) was added. The reaction pH was adjusted to 7.0 with 4.0 M aq. NaOH. The reaction was stirred at 23 °C for 3 h to give >98% HKG. HKG to malate: To the HKG-containing reaction described above, was added 184 µl (203 mg) of 30 wt% hydrogen peroxide in water (2 eq. H_2_O_2_). The reaction was stirred at 23 °C for 30 min to give >98% malate.

Malate to malonate: To the malate-containing reaction described above, was added 1.38 ml (1.53 g) of 30 wt% hydrogen peroxide in water (15 eq. H_2_O_2_). The reaction was stirred at 50 °C for 24 h to give 55% malate.

### Malonate cycle

Malonate to 3-CM: A total volume of 149 mg of sodium malonate dibasic monohydrate (0.90 mmol, 90 mM) was dissolved in 9.7 ml of a 1.0 M aq. sodium bicarbonate buffer in a 20 ml vial. Then, 298 µl (400 mg) of 50 wt% glyoxylic acid in H_2_O (3.0 eq.) was added by micropipette. The reaction pH was measured (8.4), and the vial was stirred at 50 °C for 3 h to give >98% 3-CM. Lower pH values (7.0, phosphate buffer) produced an equilibrium mixture of the aldol addition product (3-CM) and the aldol condensation product (3-carboxyfumarate, see Supplementary Table 1).

3-CM to malonate: To the 3-CM-containing reaction described above was added 1.38 ml (1.53 g) of 30 wt% hydrogen peroxide in water (15 eq. H_2_O_2_). The reaction was stirred at 50 °C for 24 h. An additional 15 eq. of hydrogen peroxide was added and the reaction was stirred for another 24 h at 50 °C to give 51% malonate.

### Malonate cycle turnover

First iteration (^13^C_1_ malonic acid to ^13^C_2_ malonic acid): A total volume of 42 mg of (2-^13^C_1_)malonic acid (0.40 mmol, 200 mM) was dissolved in 2.0 ml of 1.0 M aqueous NaHCO_3_ in a 1 dram vial. Then, 61 mg of (1,2-^13^C_2_)glyoxylic acid (2 eq.) was added, and the reaction was stirred at 50 °C for 3 h. Next, 30 wt% aqueous H_2_O_2_ was subsequently added at a rate of 40 eq./h, and the reaction was stirred at 50 °C for 10 h to produce (1,2-^13^C_2_)malonic acid.

Second iteration (^13^C_2_ malonic acid to ^13^C_3_ malonic acid): The reaction volume was reduced back to 2.0 ml under vacuum to maintain a consistent reaction concentration between iterations. Then, 61 mg of (1,2-^13^C_2_)glyoxylic acid (2 eq.) was added, and the reaction was stirred at 50 °C for 3 h. Next, 30 wt% aqueous H_2_O_2_ was subsequently added to the solution at a rate of 40 eq./h, and the reaction was stirred at 50 °C for 10 h to produce a 1:1 mixture of (1,2-^13^C_2_)malonic acid and (1,2,3-^13^C_3_) malonic acid.

### Aspartate synthesis

Glyxoylate to α-hydroxyglycine: A total volume of 5.0 g of ammonium acetate (65 mmol) in 5.0 ml of deionized (DI) water was chilled to 0 °C. Then, 3.7 ml of chilled 50 wt% glyoxylic acid in H_2_O (0.52 eq, 2.5 g) was added and the reaction was stirred at 0 °C for 45 min. The product was collected as a white precipitate and washed twice with 5 ml of cold DI water to yield >95% α-hydroxyglycine^29^.

α-Hydroxyglycine and malonic acid to β-carboxyaspartate: A total volume of 100 mg of malonic acid (0.96 mmol, 480 mM) was dissolved in 2.0 ml of 1.0 M aq. sodium bicarbonate buffer in a 1 dram vial. Then, 170 mg (2 eq.) of α-hydroxyglycine was added. The reaction was stirred at 23 °C for 5 days; the pH drifted from 7.2 to 6.8 over the reaction course. β-carboxyaspartate to aspartate: To the β-carboxyaspartate-containing reaction described above was added 195 mg of magnesium chloride hexahydrate (1 eq.), and the reaction was stirred at 60 °C for 5 h to give a two-step yield of 50% for the malonate to aspartate transformation.

### Data availability

The authors declare the data that support the findings of this study are available within the paper and its Supplementary Information files.

## Electronic supplementary material

Supplementary Information

## References

[1] Dyson, F. *Origins of Life* (Cambridge University Press, Cambridge, 1999).

[2] Wächtershäuser G (1988). Before enzymes and templates: theory of surface metabolism. Microbiol. Rev..

[3] Anet FA (2004). The place of metabolism in the origin of life. Curr. Opin. Chem. Biol..

[4] Morowitz HJ, Kostelnik JD, Yang J, Cody GD (2000). The origin of intermediary metabolism. Proc. Natl. Acad. Sci. USA.

[5] Orgel LE (2008). The implausibility of metabolic cycles on the prebiotic earth. PLoS Biol..

[6] Srinivasan V, Morowitz HJ (2009). Analysis of the intermediary metabolism of a reductive chemoautotroph. Biol. Bull..

[7] Cody GD (2000). Primordial carbonylated iron-sulfur compounds and the synthesis of pyruvate. Science.

[8] Zhang XV, Martin ST (2006). Driving parts of Krebs cycle in reverse through mineral photochemistry. J. Am. Chem. Soc..

[9] Guzman MI, Martin ST (2009). Prebiotic metabolism: production by mineral photoelectrochemistry of alpha-ketocarboxylic acids in the reductive tricarboxylic acid cycle. Astrobiology.

[10] Guzman MI, Martin ST (2010). Photo-production of lactate from glyoxylate: how minerals can facilitate energy storage in a prebiotic world. Chem. Commun..

[11] Miller SL, Smith-Magowan D (1990). The thermodynamics of the Krebs cycle and related compounds. J. Phys. Chem. Ref. Data.

[12] McMurry, J. & Begley, T. P. *The Organic Chemistry of Biological Pathways*. (Roberts and Company Publishers: Englewood, 2005). .

[13] Orgel LE (2000). Self-organizing biochemical cycles. Proc. Natl. Acad. Sci. USA.

[14] Ross DS (2007). The viability of a nonenzymatic reductive citric acid cycle - kinetics and thermochemistry. Orig. Life Evol. Biosph..

[15] Novikov Y, Copley SD (2013). Reactivity landscape of pyruvate under simulated hydrothermal vent conditions. Proc. Natl. Acad. Sci. USA.

[16] Keller MA, Turchyn AV, Ralser M (2014). Non-enzymatic glycolysis and pentose phosphate pathway-like reactions in a plausible Archean ocean. Mol. Syst. Biol..

[17] Keller MA, Kampjut D, Harrison SA, Ralser M (2017). Sulfate radicals enable a non-enzymatic Krebs cycle precursor. Nat. Ecol. Evol..

[18] Ralser M (2014). The RNA world and the origin of metabolic enzymes. Biochem. Soc. Trans..

[19] Muchowska KB (2017). Metals promote sequences of the reverse Krebs cycle. Nat. Ecol. Evol..

[20] Borda MJ, Elsetinow AR, Schoonen MA, Strongin DR (2001). Pyrite-induced hydrogen peroxide formation as a driving force in the evolution of photosynthetic organisms on an early earth. Astrobiology.

[21] Cooper G, Reed C, Nguyen D, Carter M, Wang Y (2011). Detection and formation scenario of citric acid, pyruvic acid, and other possible metabolism precursors in carbonaceous meteorites. Proc. Natl. Acad. Sci. USA.

[22] Utter MF, Keech DB (1960). Formation of oxaloacetate from pyruvate and CO2. J. Biol. Chem..

[23] Guzman MI, Martin ST (2008). Oxaloacetate-to-malate conversion by mineral photoelectrochemistry: implications for the viability of the reductive tricarboxylic acid cycle in prebiotic chemistry. Int. J. Astrobiol..

[24] Zhou R, Guzman MI (2016). Photocatalytic reduction of fumarate to succinate on ZnS mineral surfaces. J. Phys. Chem. C.

[25] Naidja A, Siffert B (1990). Oxidative decarboxylation of isocitric acid in the presence of montmorillonite. Clay. Miner..

[26] Knoop F, Martius C (1936). Über die Bildung von Citronensäure. Hoppe. Seylers. Z. Physiol. Chem..

[27] Wiley R, Kim K (1973). The bimolecular decarboxylative self-condensation of oxaloacetic acid. J. Org. Chem..

[28] Copley SD, Smith E, Morowitz HJ (2010). How life began: the emergence of sparse metabolic networks. J. Cosmol..

[29] Hoefnagel AJ, Van Bekkum H, Peters JA (1992). The reaction of glyoxylic acid with ammonia revisited. J. Org. Chem..

[30] Yamagata Y (1990). Prebiotic synthesis of orotic acid parallel to the biosynthetic pathway. Orig. Life. Evol. Biosph..

[31] Lazcano A (2009). Complexity, self-organization and the origin of life: the happy liaison?. Orig. Life Self-Organization Biol. Evol..

[32] Mohammed FS (2017). A plausible prebiotic origin of glyoxylate: nonenzymatic transamination reactions of glycine with formaldehyde. Synlett.

[33] Warneck P (2005). Multi-phase chemistry of C2 and C3 organic compounds in the marine atmosphere. J. Atmos. Chem..

[34] Wang W, Qu Y, Yang B, Liu X, Su W (2012). Lactate oxidation in pyrite suspension: a Fenton-like process in situ generating H 2O 2. Chemosphere.

[35] Encrenaz T, Greathouse TK, Lefevre F, Atreya SK (2012). Hydrogen peroxide on Mars: observations, interpretation and future plans. Planet Space Sci..

[36] Carlson RW (1999). Hydrogen peroxide on the surface of Europa. Science.

[37] Liang MC, Hartman H, Kopp RE, Kirschvink JL, Yung YL (2006). Production of hydrogen peroxide in the atmosphere of a Snowball Earth and the origin of oxygenic photosynthesis. Proc. Natl. Acad. Sci. USA.

[38] Mendoza AN, Ponnamperuma C (1982). Prebiotic formation of higher molecular weight compounds from the photolysis of aqueous acetic acid. Photochem. Photobiol..

[39] Rice GB, Yerabolu JR, Krishnamurthy R, Springsteen G (2017). The abiotic oxidation of organic acids to malonate. Synlett.

[40] Zubay G (2003). The glyoxylate cycle, a possible evolutionary precursor of the TCA cycle. Chemtracts.

[41] Eschenmoser A (2007). The search for the chemistry of life’s origin. Tetrahedron.

