# Supplementary material for: Linked cycles of oxidative decarboxylation of glyoxylate as protometabolic analogs of the citric acid cycle
Source: Nat Commun. 2018 Jan 8;9:91. doi: 10.1038/s41467-017-02591-0 (PMC5758577; doi:10.1038/s41467-017-02591-0)
Supplement: Supplementary file 1 — Supplementary Information [file 41467_2017_2591_MOESM1_ESM.pdf]

Supplementary Methods

Materials:

The following reagents were purchased and used as received: oxaloacetic acid (≥ 97%, Sigma-Aldrich O4126), DL-malic acid (≥99%, Sigma-Aldrich 240176), sodium malonate dibasic monohydrate (Sigma-Aldrich M4795), 50 wt% in H_2O glyoxylic acid solution, 30 wt% in H_2O hydrogen peroxide solution (contains inhibitor, assayed 29.0 to 32.0%, Sigma-Aldrich 216763), sodium pyruvate (≥99%, Sigma-Aldrich P2256), malonic acid (≥99%, Sigma-Aldrich M1296). (1,2-$^{13}C_2$)glyoxylic acid (contaminated with 3% glycolic acid, and 1.5% oxalic acid), and (2-$^{13}C_1$)malonic acid (99%) were purchased from Cambridge Isotope Laboratories, Inc., and used as received:.

Procedures:

HKG cycle

Oxaloacetate to HKG: 119 mg of oxaloacetic acid (0.90 mmol, 90 mM) was dissolved in 9.9 ml of a 1.0 M aq. pH 7 sodium phosphate buffer in a 20-ml vial (Chemglass, CG-4912-05) with a small stirbar. 119 µl (160 mg) of 50 wt% glyoxylic acid in H_2O (1.2 eq.) was added by micropipette. The reaction pH was adjusted to 7.0 with 4.0 M aq. NaOH as measured by a calibrated Mettler Toledo InLab Expert Pro probe. The reaction vial was placed on a magnetic stir plate (Ika, RCT basic) fitted with a vial reaction block heater (ChemGlass, CG-1991-01) and stirred at 23 °C for 3 h to give >98% HKG. *oxalomalate (OXM):* ^{1}H NMR, 400 Mz, D_2O – *diastereomer A:* δ 4.27 ppm (1H, d), δ 4.32ppm (1H, d), *diastereomer B* - δ 4.28 ppm (1H, d), δ 4.34 ppm (1H, d). *4-hydroxy-2-ketoglutarate (HKG):* ^{1}H NMR, 400 Mz, D_2O – δ 2.90 ppm (1H, dd), δ 3.05 ppm (1H, dd), δ 4.25 ppm (1H, dd)

HKG to malate: To the HKG-containing reaction described above, was added 184 µl (203 mg) of 30 wt% hydrogen peroxide in water (2 eq. H_2O_2). The reaction was stirred at 23 °C for 30 min to give >98% malate. *malate:* ^{1}H NMR, 400 Mz, D_2O – δ 2.20 ppm (1H, dd), δ 2.50 ppm (1H, dd),δ 4.15 ppm (1H, dd)

Malate to malonate: To the malate-containing reaction described above, was added 1.38 ml (1.53 g) of 30 wt% hydrogen peroxide in water (15 eq. H_2O_2). The reaction was stirred at 50 °C for 24 h to give 55% malate. *malonate:* ^{1}H NMR, 400 Mz, D_2O – δ 2.93 ppm (2H, s), ^{13}C NMR, 400 Mz, D_2O – δ 42 ppm

Additional Notes: The aldol addition (to generate OXM), the decarboxylation (to generate HKG), and the oxidation (to generate malonate) are tolerant to buffer type (phosphate and carbonate were employed) and pH values (5.5 to 10.5 were employed), see Supplementary Figs. 4–6. The oxidation reaction of malate to malonate is accelerated by the inclusion of iron-based Lewis acids (ferrous sulfate, ferric sulfate, iron disulfide), see Supplementary Table S1.

Malonate cycle

Malonate to 3-carboxymalonate (3-CM): 149 mg of sodium malonate dibasic monohydrate (0.90 mmol, 90 mM) was dissolved in 9.7 ml of a 1.0 M aq. sodium carbonate buffer in a 20-ml vial. 298 µl (400 mg) of 50 wt% glyoxylic acid in H_2O (3.0 eq.) was added by micropipette. The reaction pH was measured (8.4), and the vial was placed in a heat block and stirred at 50 °C for 3 h to give >98% 3-CM. Lower pH values (7.0, phosphate buffer) produced an equilibrium mixture of the aldol addition product (3-CM) and the aldol condensation product (3-carboxyfumarate, 3-CF, see Table S1). *3-carboxymalate (3-CM):* ^{1}H NMR, 400 Mz, D_2O – δ 3.45 ppm (1H, d), δ 4.22 ppm (1H, d), *3-carboxyfumarate (3-CF):* ^{1}H NMR, 400 Mz, D_2O – δ 6.17 ppm (1H, s)

3-carboxymalonate (3-CM) to malonate: To the 3-CM-containing reaction described above, was added 1.38 ml (1.53 g) of 30 wt% hydrogen peroxide in water (15 eq. H_2O_2). The reaction was stirred at 50 °C for 24 h. An additional 15 eq. of hydrogen peroxide was added and the reaction was stirred for another 24 h at 50 °C to give 51% malonate.

Malonate Cycle Turnover

1st iteration ($^{13}C_1$ malonic acid to $^{13}C_2$ malonic acid): 42 mg of (2-$^{13}C_1$)malonic acid (0.40 mmol, 200 mM) was dissolved in 2.0 ml of 1.0 M aqueous $NaHCO_3$ in a 1-dram vial. 61 mg of (1,2-$^{13}C_2$)glyoxylic acid (2 eq.) were added and the reaction pH was measured (8.0). The reaction was stirred at 50 °C for 3 h. 30 wt% aqueous H_2O_2 was subsequently added at a rate of 40 eq/h, and the reaction was stirred at 50 °C for 10 h to produce (1,2-$^{13}C_2$)malonic acid (Calcd. [M-H] m/z: 105.0098, Found: 105.0020).

2nd iteration ($^{13}C_2$ malonic acid to $^{13}C_3$ malonic acid): The reaction volume was reduced back to 2.0 ml under vacuum to maintain a consistent reaction concentration between iterations. 61 mg of (1,2-$^{13}C_2$)glyoxylic acid (2 eq.) were added and the reaction pH was measured (8.0). 30 wt% aqueous H_2O_2 was added to the solution at a rate of 40 eq/h, and the reaction was stirred at 50 °C for 10 h to produce a 1:1 mixture of (1,2-$^{13}C_2$)malonic acid and (1,2,3-$^{13}C_3$) malonic acid (Calcd. [M-H] m/z: 106.0132, Found: 106.0033).

Pyruvate to HKG

149 mg of sodium pyruvate (0.90 mmol, 90 mM) was dissolved in 9.7 ml of 1.0 M aq. sodium bicarbonate buffer in a 20-ml vial. 149 µl (200 mg) of 50 wt% glyoxylic acid in H_2O (1.5 eq.) was added by micropipette. The reaction pH was measured at 7.9. The reaction was then placed in a heat block and stirred at 50 °C for 1 h.

Aspartate Synthesis

glyxoylate to α-hydroxyglycine: 5.0 g of ammonium acetate (65 mmol) in 5.0 ml of deionized water was chilled to 0 °C. 3.7 ml of chilled 50 wt% glyoxylic acid in H_2O (0.52 eq, 2.5 g) was added and the reaction was stirred at 0 °C for 45 min. The product was collected as a white precipitate and washed twice with 5 ml of cold deionized water to yield >95% α-hydroxyglycine *(15). α-hydroxyglycine* : ^{13}C NMR, 400 Mz, D_2O – δ 87 ppm, 176 ppm.

α-hydroxyglycine and malonic acid to β-carboxyaspartate: 100 mg of malonic acid (0.96 mmol, 480 mM) was dissolved in 2.0 ml of 1.0 M aq. sodium bicarbonate buffer in a 1-dram vial. 170 mg (2 eq.) of α-hydroxyglycine was added. The reaction was stirred at 23 °C for 5 d; the pH drifted from 7.2 to 6.8 over the reaction course. *β-carboxyaspartate:* ^{13}C NMR, 400 Mz, D_2O – δ 59 ppm, 74 ppm, 173 ppm, 173 ppm, 174 ppm. Mass Spec. MH+ 178.03

β-carboxyaspartate to aspartate: To the β-carboxyaspartate-containing reaction described above, was added 195 mg of magnesium chloride hexahydrate (1 eq.), and the reaction was stirred at 60 °C for 5 h to give a two-step yield of 50% for the malonic acid to aspartate transformation. *aspartate:* ^{1}H NMR, 400 Mz, D_2O – δ 2.55 ppm (1H, dd), δ 2.68 ppm (1H, dd), δ 3.76 ppm (1H, dd). Mass Spec. MH+ 134.04

Supplementary Figures

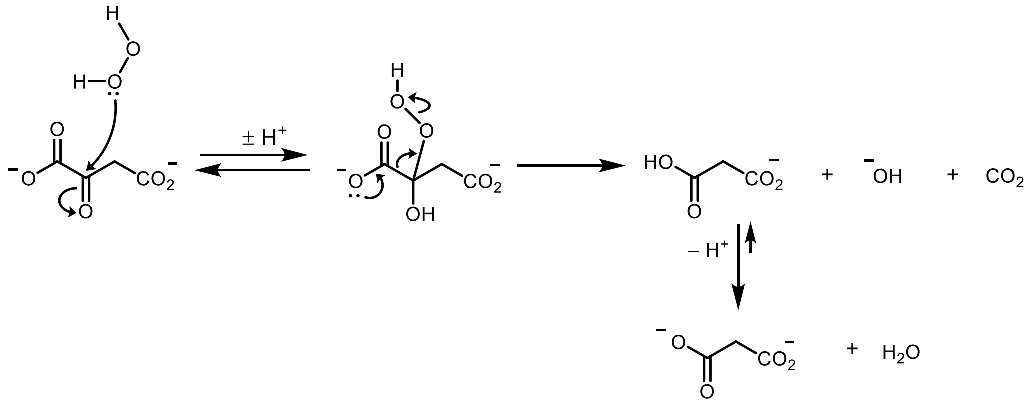

Supplementary Figure 1. The electron-moving mechanism of oxidative decarboxylation of oxaloacetate to produce malonate.

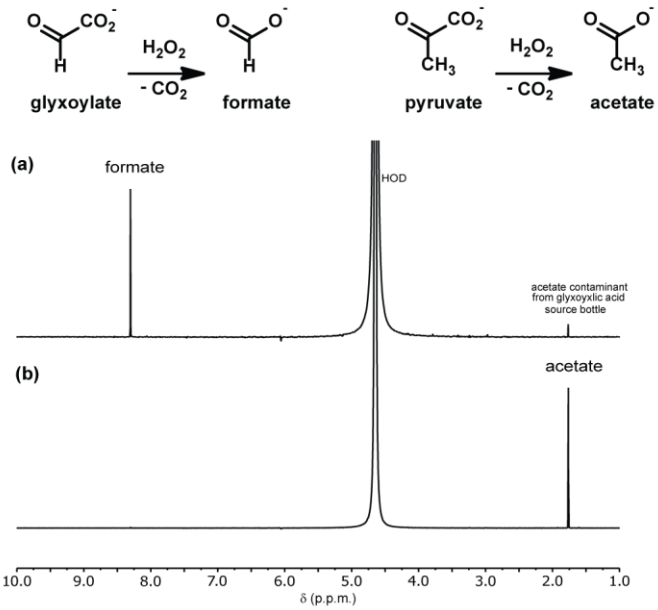

Supplementary Figure 2. Oxidation of glyoxylate and pyruvate by H_2O_2 to formate and acetate respectively. (**a**) 100 mM glyoxylate, 2 eq. H_2O_2, pH 7, 1 M phosphate buffer, 15 min, 23 °C, (**b**) 100 mM pyruvate, 2 eq. H_2O_2, pH 7, 1 M phosphate buffer, 15 min, 23 °C.

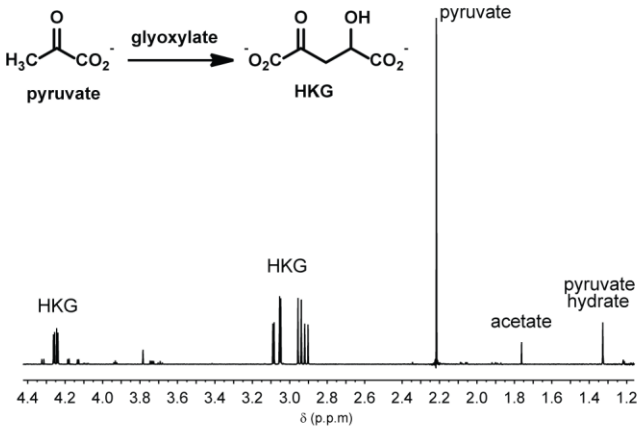

Supplementary Figure 3. Entry into the protometabolic cycles from pyruvate. 90 mM pyruvate, 1.5 eq. glyoxylate, in 10 ml of 1.0 M aq. sodium bicarbonate at pH 7.9, was heated at 50 °C for 1 h to produce HKG in 65% yield.

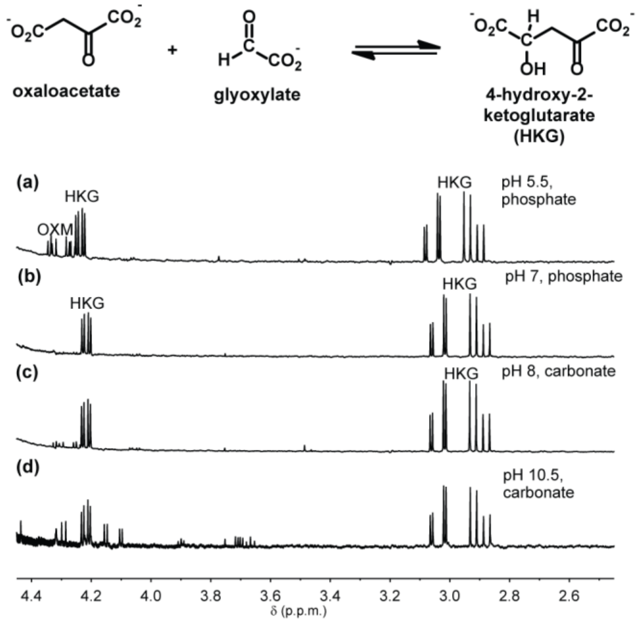

Supplementary Figure 4. The pH and buffer dependence of the aldol addition of oxaloacetate to glyoxylate in the HKG cycle. 90 mM oxaloacetate with 1.5 eq. of glyoxylic acid at 23 °C for 100 min, (**a**) pH 5.5 in 1.0 M sodium phosphate, (**b**) pH 7.0 in 1.0 M sodium phosphate, (**c**) pH 8.0 in 1.0 M sodium bicarbonate, (**d**) pH 10.5 in 1.0 M sodium carbonate.

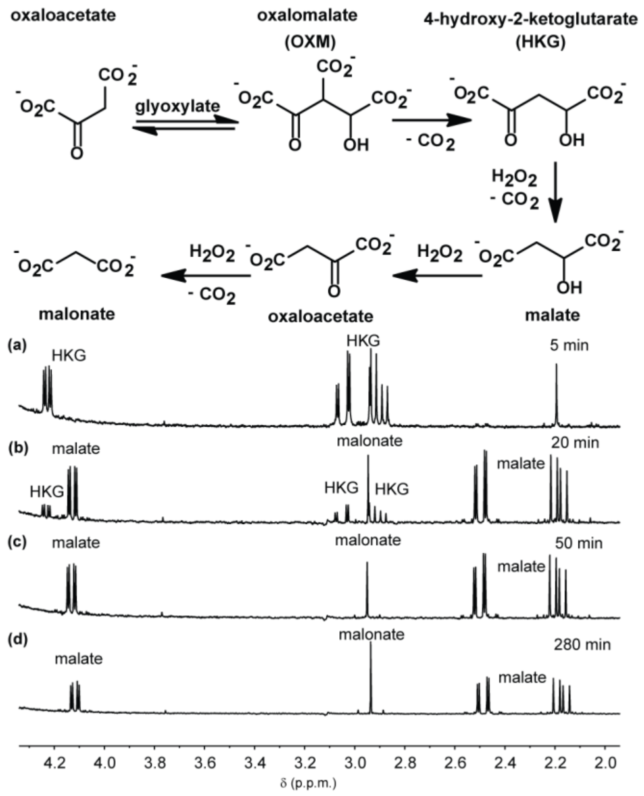

Supplementary Figure 5. Progression of the HKG cycle from oxaloacetate to malate, and then on to malonate in carbonate buffer. 90 mM oxaloacetate in 1.0 M $NaHCO_3$, 1.5 eq. of glyoxylic acid, pH 7.2 to 8.2, 52 °C. 30 wt% hydrogen peroxide was added at a rate of 5 eq./h. ^{1}H NMR at (**a**) 5 min, (**b**) 20 min, (**c**) 50 min, (**d**) 280 min.

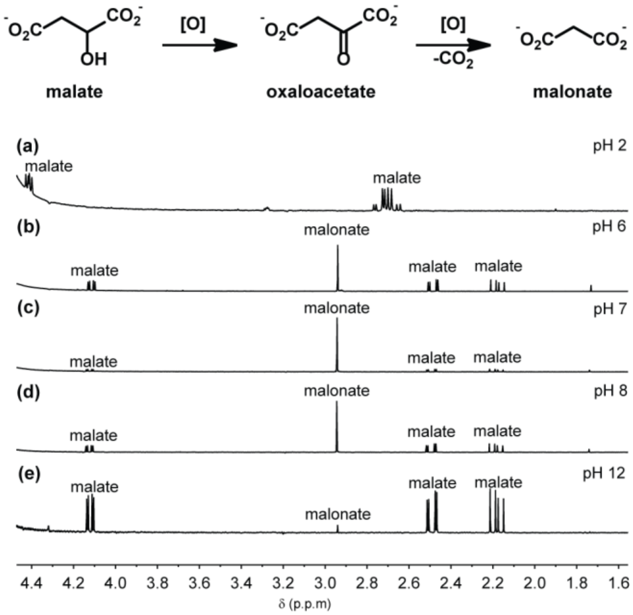

Supplementary Figure 6. The pH dependence of the oxidation of malate to malonate. 90 mM malate in 1.0 M sodium phosphate, 30 eq. H_2O_2 at once, 50 °C for 24 h at (**a**) pH 2, (**b**) pH 6, (**c**) pH 7, (**d**) pH 8, (**e**) pH 12.

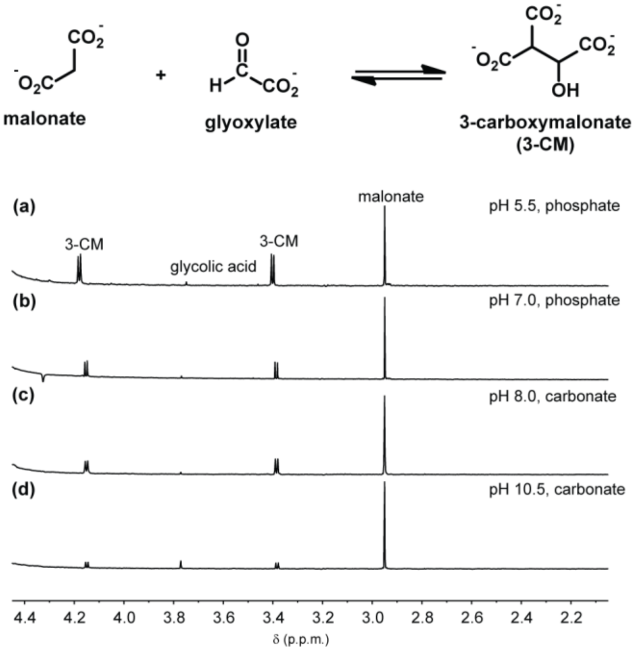

Supplementary Figure 7. The pH and buffer dependence of the aldol addition of malonate to glyoxylate in the Malonate cycle. 90 mM sodium malonate with 3 eq. of glyoxylic acid at 23 °C for 48 h. (**a**) pH 5.5 in 1.0 M sodium phosphate (**b**) pH 7.0 in 1.0 M sodium phosphate, (**c**) pH 8.0 in 1.0 M sodium bicarbonate, (**d**) pH 10.5 in 1.0 M sodium carbonate. All reactions adjusted to initial pH with 2 M NaOH. Final reaction pH values at 48 h were 5.4, 7.0, 8.3, and 10.7 respectively.

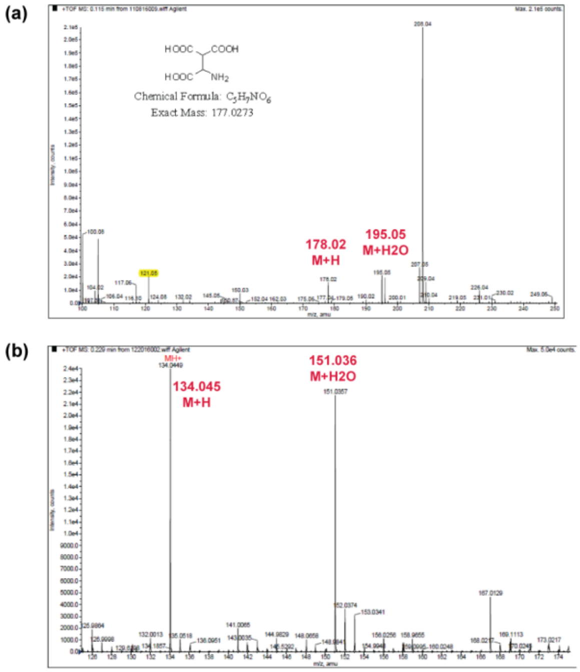

Supplementary Figure 8. Aspartate synthesis from malonate and α-hydroxyglycine. (**a**) +TOF MS of 480 mM malonic acid, with 2 eq. of α-hydroxyglycine in 1.0 M sodium bicarbonate buffer at 23 °C for 5 d generates β-carboxyaspartate (178.03). (**b**) Subsequent addition of 1 eq. of $MgCl_2$ and heating at 60 °C for 5 h produces aspartate (134.04).

Supplementary Table 1: Reaction conditions and yields for HKG and Malonate cycle reactions.

Reaction Step	Reaction Scheme	Product Yield[i]	SM	Reaction Conditions
**HKG Cycle**				
oxaloacetate to HKG	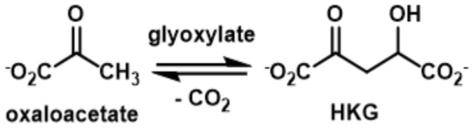	>98%	<2%	90 mM oxaloacetic acid, 1.2 eq. glyoxylate, pH 7 in 1 M phosphate buffer for 3 h at 23 °C
		>98%	<2%	90 mM oxaloacetic acid, 1.2 eq. glyoxylate, pH 8 in 1 M carbonate buffer for 3 h at 23 °C
HKG to malate	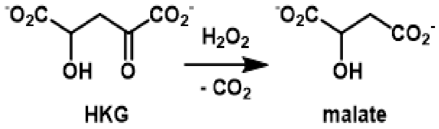	>98%	<2%	90 mM HKG, 2 eq. H_2O_2, pH 7, 1 M phosphate buffer for 30 min at 23 °C
		>98%	<2%	90 mM HKG, 2 eq. H_2O_2, pH 8 1 M carbonate buffer for 30 min at 23 °C
malate to malonate	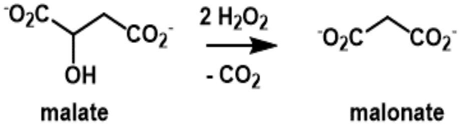	55%	14%	90 mM malic acid, 15 eq. H_2O_2, pH 7 in 1 M phosphate buffer for 24 h at 50 °C
		57%	4%	90 mM malic acid, 15 eq. H_2O_2, pH 7 in 1 M phosphate buffer for 3 h at 50 °C, with 0.25 eq. of ferrous sulfate
		52%	16%	90 mM malic acid, 15 eq. H_2O_2, pH 8 in 1 M carbonate buffer for 3 h at 50 °C, with 0.25 eq. of ferrous sulfate
**pyruvate to HKG**	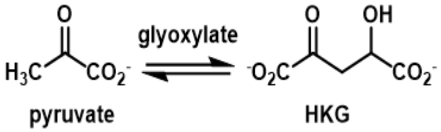	65%	30%	90 mM pyruvate, 1.5 eq. of glyoxylic acid, pH 7.9 in 1 M bicarbonate buffer for 1 h at 50 °C.
**malonate to aspartate**	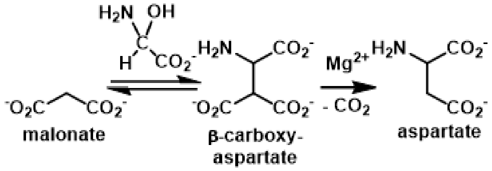	50%		480 mM malonic acid, 2 eq. α-hydroxyglycine, pH 7 in 1 M bicarbonate buffer for 5 days at 23 °C. Subsequently, 1 eq. $MgCl_2$ was added and the reaction was heated for 5 h at 60 °C

Supplementary Table 1 continued

Reaction Step	Reaction Scheme	Product Yield[i]	SM[i]	Reaction Conditions
**Malonate Cycle**				
oxaloacetate to malonate	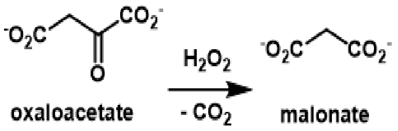	>98%	<2%	90 mM oxaloacetic acid, 1.2 eq. H_2O_2, pH 7 in 1 M phosphate buffer for 30 min at 23 °C
		>98%	<2%	90 mM oxaloacetic acid, 1.2 eq. H_2O_2, pH 8 in 1 M phosphate buffer for 30 min at 23 °C
malonate to 3-CM	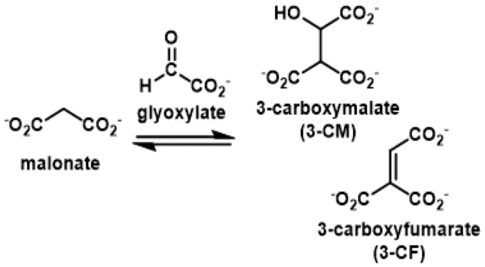	71% (+ 26% 3-CF)	3%	90 mM malonate, 3 eq. of glyoxylic acid, pH 7 in 1 M phosphate buffer for 8 h at 50 °C
		>98%	<2%	90 mM malonate, 3 eq. of glyoxylic acid, pH 8.4 in 1 M bicarbonate buffer for 24 h at 50 °C
3-CM to malonate	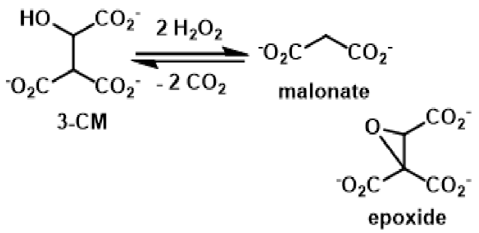	21% (+ 74% epoxide)	<1%	90 mM malic acid, 15 eq. H_2O_2, pH 7, in 1 M phosphate buffer for 24 h at 50 °C
		36%	48%	90 mM malic acid, 15 eq. H_2O_2, pH 8, in 1 M bicarbonate buffer for 48 h at 50 °C
		51%	49%	90 mM malic acid, 15 eq. H_2O_2, + 15 eq. H_2O_2 at 24 h, pH 8 in 1 M bicarbonate buffer for 48 h total at 50 °C

i. Product yields and remaining starting material (SM) were measured by ^{1}H NMR integration compared with an internal standard (*t*-butanol) in the D_2O NMR solvent. Yields are calculated as the percent of the theoretical yield from limiting reactant (non-isolated).
